# Age as an Effect Modifier of the Effects of Transcutaneous Auricular Vagus Nerve Stimulation (taVNS) on Heart Rate Variability in Healthy Subjects

**DOI:** 10.3390/jcm13144267

**Published:** 2024-07-22

**Authors:** Anna Carolyna Gianlorenço, Kevin Pacheco-Barrios, Marianna Daibes, Lucas Camargo, Hyuk Choi, Jae-Jun Song, Felipe Fregni

**Affiliations:** 1 Laboratory of Neuroscience and Neurological Rehabilitation, Physical Therapy Department, Federal University of Sao Carlos, Sao Carlos 13565-905, SP, Brazil; gianlorenco@ufscar.br; 2 Neuromodulation Center, Center for Clinical Research Learning, Spaulding Rehabilitation Hospital, Massachusetts General Hospital, Harvard Medical School, 1575 Cambridge Street, Cambridge, MA 02139, USA; kevin.pacheco.barrios@gmail.com (K.P.-B.); mdaibesrachiddeandrade@mgb.org (M.D.); lcamargo@mgb.org (L.C.); 3 Department of Medical Sciences, Graduate School of Medicine, Korea University, Seoul 02841, Republic of Korea; hyuk76@korea.ac.kr; 4 Neurive Co., Ltd., Gimhae 08308, Republic of Korea; jjsong23@gmail.com; 5 Department of Otorhinolaryngology-Head and Neck Surgery, Korea University Medical Center, Seoul 02841, Republic of Korea

**Keywords:** taVNS, heart rate variability, vagus nerve, transcutaneous auricular vagus nerve stimulation

## Abstract

**Background**: Evidence suggests that vagus nerve stimulation can modulate heart rate variability (HRV). However, there is a lack of mechanistic studies in healthy subjects assessing the effects of bilateral transcutaneous auricular vagus nerve stimulation (taVNS) on HRV. Our study aims to investigate how taVNS can influence the HRV response, including the influence of demographic variables in this response. **Methods**: Therefore, we conducted a randomized controlled study with 44 subjects, 22 allocated to active and 22 to sham taVNS. **Results**: Our results showed a significant difference between groups in the high-frequency (HF) metric. Active taVNS increased the HF metric significantly as compared to sham taVNS. Also, we found that age was a significant effect modifier of the relationship between taVNS and HF-HRV, as a larger increase in HF-HRV was seen in the older subjects. Importantly, there was a decrease in HF-HRV in the sham group. **Conclusions**: These findings suggest that younger subjects can adapt and maintain a constant level of HF-HRV regardless of the type of stimulation, but in the older subjects, only the active taVNS recipients were able to maintain and increase their HF-HRV. These results are important because they indicate that taVNS can enhance physiological regulation processes in response to external events.

## 1. Introduction

Invasive Vagus Nerve Stimulation (iVNS) is an approved treatment for refractory epilepsy [1,2,3], depression [4,5], and stroke [6,7,8]. However, despite its effectiveness, it is an invasive procedure with substantial costs, and only around 30% of implanted patients exhibit a clinical response [9]. Transcutaneous auricular vagus nerve stimulation (taVNS), as an alternative, is a safe and non-invasive stimulation technique that targets the auricular branch of the vagus nerve, placing the electrodes at the concha or tragus of the ear. Several studies, including reviews [6,7,8] and meta-analyses [2], have suggested that taVNS has potential applications in treating different conditions, including epilepsy [10], depression [11,12], anxiety [13], chronic pain [14,15], and stroke [16], with observed anti-inflammatory and immunomodulating effects [6].

It has also been demonstrated that taVNS can affect several brain functions by stimulating the vagal afferents. From the vagus nerve, inputs are sent to the nucleus of the solitary tract (NTS), which has connections to the locus coeruleus (LC), a major adrenergic center [17]. The locus coeruleus has projections to several cortical and subcortical structures, including the frontal and parietal cortex, thalamic nucleus, and hippocampus [18]. Together with integrating and processing central impulses, some of these brain areas also control the vagal efferents. Although there is a lack of data regarding taVNS’s effects on the vagal efferent, it could be an intriguing non-invasive intervention with potential to affect the vagal efferent functions [19].

One important influence of the vagus nerve efferent function is on the modulation of heart rate through sinoatrial node activity. Heart rate variability (HRV) is a non-invasive way to measure the vagal influence by measuring the variability in time between successive heartbeats [19]. Given this role, HRV has been viewed and investigated as a major biomarker for neurocardiac function, with links to stress, illness, and mortality, as well as an individual’s state of health [19,20]. Considering that decreased HRV has been related to morbidity and mortality of different diseases through mechanisms such as imbalance of the autonomic nervous system (ANS), inflammatory response, and oxidative stress, restoring this equilibrium through taVNS could have a therapeutic effect [20,21].

Previous trials have shown and discussed how taVNS affects HRV [22,23], suggesting that the stimulation can induce a shift toward greater parasympathetic tonus. One important concept is that the parasympathetic tonus is dependent on several factors such as (i) age, (ii) diseases, and (iii) physiological states (e.g., no movement/resting vs. during movement). The physiological mechanisms of heart rate dynamics can be explained by (i) respiratory gating, which determines the high frequency (HF) (0.18–0.40 Hz) associated with vagal (parasympathetic) activity and central blood pressure [24,25]; (ii) sympathetic vasomotor activity that determines the low frequency (LF) (0.03–0.15 Hz) associated with reflex baroreceptor activity [26]; and sympathovagal balance, which can be measured by the LF/HF ratio [27].

Normal aging is known to be related to decreased global autonomic regulation, which results in a decreased HRV [28,29]. Umetani et al. (1998) demonstrated that healthy subjects older than 65 years old have deterioration in the standard deviation of the R-R intervals (SSDR), the root mean square of the difference between successive R-R intervals (RMSSD), and the percentage of R-R intervals, which differed by more than 50 ms (pNN50) from values in subjects younger than 30 years old [30]. Therefore, given that ANS activity represents an important interface and marker for regulating physiological processes according to a neural response to external events, understanding this marker provides important insights about how the brain responds during normal aging and pathological conditions, such as neurological disorders.

Given the importance of HRV markers and the preliminary results showing that taVNS can regulate HRV, we aimed to understand how bilateral taVNS modulates HRV in subjects in a resting state and, in addition, understand whether these effects are modified by demographic characteristics of these individuals, such as age and sex.

## 2. Methods

### 2.1. Study Design

In this randomized, double-blind, sham–control trial, we recruited 44 healthy subjects who were allocated into two groups (allocation ratio 1:1): active taVNS or sham taVNS. The study was conducted according to the guidelines of the Declaration of Helsinki and approved by the Institutional Review Board of the Partners Human Research Committee (protocol number: 2022P003200; approved on 4 June 2023). Informed consent was obtained from all subjects involved in the study according to the Declaration of Helsinki (1964). This clinical trial was registered in ClinicalTrials.gov (NCT05801809).

### 2.2. Participants

This study included healthy subjects older than 18 and naïve to stimulation with taVNS. We excluded patients with any unstable medical condition, history of alcohol or drug abuse in the past 6 months, or the presence of any contraindication to taVNS, such as implanted cranial or cardiac devices or metal in the cranium, and we excluded subjects with a score higher than 30 in the Beck Depression Inventory (BDI), which indicates severe depression. All participants signed an online consent form in an encrypted web-based platform (REDCap).

### 2.3. Intervention

**Figure 1:**
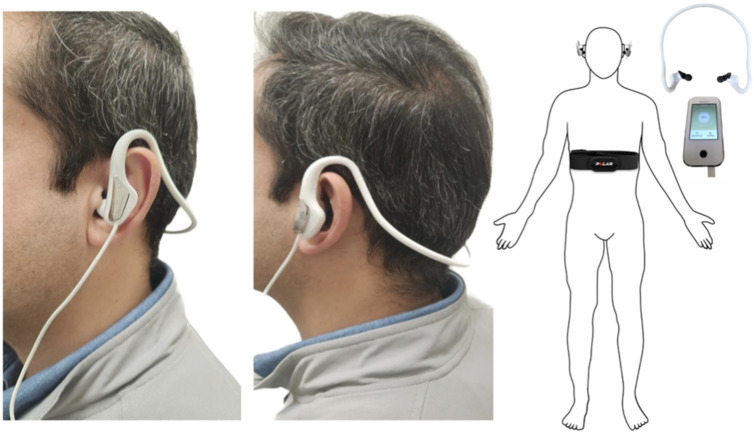
A representation of a subject receiving taVNS bilaterally.

We used a Healaon taVNS device (Neurive Inc., Gimhae, Republic of Korea), which consists of an ear set with conductive ear tips placed on the auricular concha of the ears, displayed in Figure 1. Carbon polymer electrodes were connected to a stimulator, and during active stimulation, both cymba conchae of the auricular were stimulated at 30 Hz, 200–250 us, with adjustable intensity below the pain threshold (~1.5 mA), for 60 min (Supplementary Table S1). The sham intervention was applied at the same location bilaterally to the cymba conchae of the auricular, but the device did not provide stimulation during the 60 min. The active and sham devices were identical visually, and an individual not involved in data collection and analysis followed the randomization to hand the devices to the experimenter, allowing for blindness.

### 2.4. Outcomes

In this study, we investigated the effects of a single active taVNS session on various indices of HRV compared to a sham. A short-term HRV register (5 min in a sitting position) was recorded pre- and post-taVNS intervention.

To collect heart rate variability (HRV) data, we used the H10 Polar device to make recordings through the validated HRV4Training app on an Android device; we followed the standard recommended settings [31,32,33]. The process involved first ensuring proper placement of the H10 Polar device on the chest, securely fastening it for accurate readings. Data were transmitted in real time using Bluetooth connectivity; the device began recording HRV metrics continuously.

Regarding the frequency-domain metrics, which were based on divisions by the Task Force of the European Society of Cardiology and the North American Society of Pacing and Electrophysiology (1996) [34], we examined the high frequencies (HFs), low frequencies (LFs), and LF/HF ratio. We also explored a non-linear method to calculate the short-term detrended fluctuation exponent (DFA-alpha1). The LF captures the magnitude of heart oscillations in the range of three to nine cycles per minute (0.04 to 0.15 HZ) and is assumed, yet debatable [35], to have a dominant sympathetic component, while the HF captures heart rate oscillations of 9 to 24 cycles per minute (0.15 to 0.40 Hz) and is assumed to mirror the parasympathetic tonus [19]. Their ratio is discussed elsewhere [36]. While some sources describe it as a sympathovagal balance, there are others who argue that the LF is not exclusively representative of the sympathetic activity, together with a non-linear interaction between both autonomic systems, challenging the assumption that the LF/HF ratio accurately reflects this balance.

The selection of time-domain metrics, which highlight variability in the interbeat interval (IBI) between successive heartbeats, is grounded in their established correlation with autonomic nervous system activity, as demonstrated by Parati et al. (1995) [37]. We therefore assessed the following parameters: encompassed parameters such as the standard deviation of the N-N intervals (SDNN), counts of R-R intervals differing by more than 50 ms (NN50), the percentage of NN50 (pNN50), and the root mean square of the successive differences in R-R intervals (RMSSD).

We present these results after extraction from the built-in HRV analysis module of the HRV4training app. All values were reported and used as raw metrics, except for LF and HF metrics, where normalized units were employed [38]. It is noteworthy to mention that employing normalized units for LF and HF has distinct advantages in result interpretation. These units offer a more standardized and reliable comparison across different individuals and populations, providing insights into autonomic nervous system activity that might be obscured when using raw values alone due to high intra- and inter-subject variability [38].

In addition, we collected demographic data from all subjects enrolled in this study. Those variables include age (as continuous and binary, with 60 years being the cut off), biological sex, and education level, which we used as covariates.

### 2.5. Sample Size

We planned the sample size using the available data from previous studies on taVNS and HRV [22,39]. The effect sizes ranged from 1.01 (changes in total HRV power) to 13.72 (changes in HF-HRV). We could detect a variety of scenarios and different magnitudes of sample sizes, and we decided to choose a more conservative approach (effect size of 1.01). We assumed a type I error of 5% (alpha), a type II error of 20% (beta), and a power of 80% in a two-sided *t*-test for independent samples, and we estimated that a sample of 36 subjects would be needed. However, we expanded the sample by 20% to account for a conservative attrition rate and to increase power for secondary outcome analysis. Therefore, we included a total sample size of 44 subjects, leading to 22 subjects in each group.

### 2.6. Randomization and Masking

Using a web-based program (http://randomization.com), we created a randomization list in a 1:1 allocation ratio and kept it in sealed envelopes. An uninvolved staff member placed the randomization order in numbered sealed envelopes and maintained it throughout the study. Therefore, we could keep the blinding for participants and co-investigators, including outcome assessors.

### 2.7. Statistical Analysis

The statistical analyses were carried out with STATA version 18. The differences between the metrics at baseline and after stimulation were calculated. A histogram was used to assess data distribution. Baseline characteristics were reported using descriptive statistics (e.g., mean and SD for continuous variables, frequency tabulations for categorical variables) for each group and compared using an independent *t*-test for continuous variables and Fisher’s exact test for categorical and binary variables.

The statistical analysis was focused on evaluating the impact of group assignment on various HRV outcomes, listed above. We first tested differences between groups by an independent *t*-test. Additionally, to calculate the adjust effects, we employed linear regression models to test for differences between the treatment and control groups across the outcome variables and to account for important covariates such as age and biological sex. The difference in scores (post-taVNS minus pre-taVNS) for each outcome was the dependent variable, and the groups (active and placebo) were the independent variable. The results for these variables were adjusted for the interaction term using the covariates mentioned above, such as group*age_group. However, only the interaction term (group*age_group) significantly altered the regression.

For the models, we evaluated the linearity assumption by visually examining the scatterplot of each independent variable alongside a superimposed regression line plot. The assumption of homoscedasticity was verified through visual inspection of the scatterplot depicting standardized predicted values against standardized residuals [40]. We assessed the normality of residuals through histograms and the Shapiro–Wilk normality test. Additionally, the central limit theorem was applied to distributions that approximated a normal distribution [41]. To assess the relationships between changes (post-intervention minus baseline) in HRV indices, we conducted a Pearson correlation analysis. A significance level of *p* < 0.05 was set a priori to determine the statistical significance.

## 3. Results

### 3.1. Healthy Participants

The characteristics of the patients at baseline were well balanced between both groups. The mean age was 41.3 years and 61.3% of the sample were women (Table 1). For the HRV metrics, there was some missing data from movement artifacts and intermittent signal loss in consecutive RR intervals, resulting in a sample ranging between 33 and 37 subjects. Independent sample *t*-tests for continuous variables and chi-squared tests or Fisher’s exact tests for binary and categorical variables, when comparing proportions, revealed no significant differences between baseline measures, with *p*-values ranging from 0.22 to 0.88.

### 3.2. Changes in HRV

**Figure 2:**
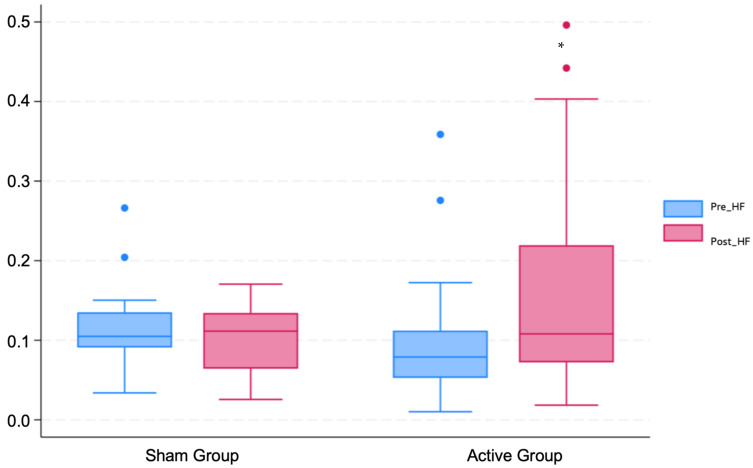
Descriptive boxplots of pre-intervention (Pre_HF) and post-intervention (Post_HF) high-frequency (HF) heart rate variability for the sham group and active group. * *p* < 0.05.

**Figure 3:**
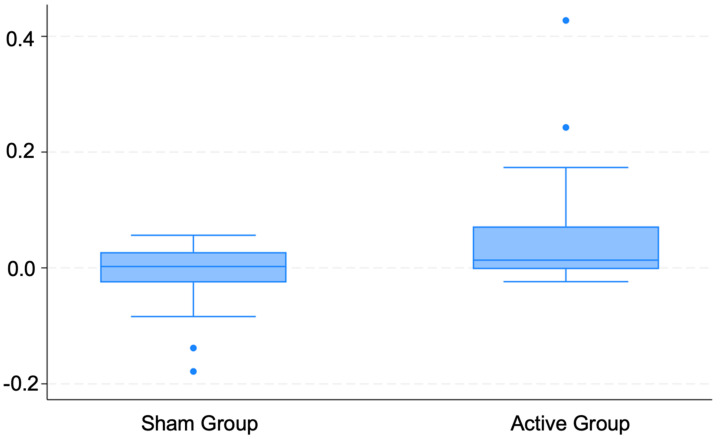
Descriptive boxplot showing the delta in high-frequency (ΔHF) heart rate variability from baseline to post-intervention for the sham group versus the active group. The *Y*-axis quantifies the change (final − baseline) in HF metrics.

When looking at the differences between groups, there is a significant difference in the HF metric following the active stimulation of the vagus nerve as compared to sham taVNS. Active taVNS increased the HF-HRV significantly (t(36): 2.4, *p* = 0.02). Figure 2 and Figure 3 are a descriptive illustration of the changes in heart rate variability—HF metric—for both groups (Sham taVNS and Active taVNS) before (Pre_HF) and after (Post_HF) the stimulation, showing the pronounced increase in HF-HRV in the active group with the stimulation. This highlights the influence of taVNS on the HF component, indicating a shift in the cardiac autonomic function towards parasympathetic predominance.

In contrast, all other metrics of HRV measured in the trial (with their baseline values described in Table 1) did not reveal any significant changes between the active and sham groups in the LF/HF ratio (mean increase: sham 0.033 vs. active taVNS 0.218; t(36) = 1.25, *p* = 0.21) and SDNN (mean increase: sham 13.57 vs. active taVNS 6.62; t(36) = −0.77, *p* = 0.44). The RMSSD metric increased in the sham group (4.96) vs. a decrease in the active group (−4.79) (t(36) = −1.29, *p* = 0.20); PNN50 had an increase in the sham group of 2.33 vs. a decrease in active taVNS of −2.79 (t(32) = −1.23, *p* = 0.22); and HR had a mean decrease in sham of −2.23 vs. active taVNS of −2.77 (t(36) = −0.33, *p* = 0.73).

### 3.3. Regression Analysis

**Figure 4:**
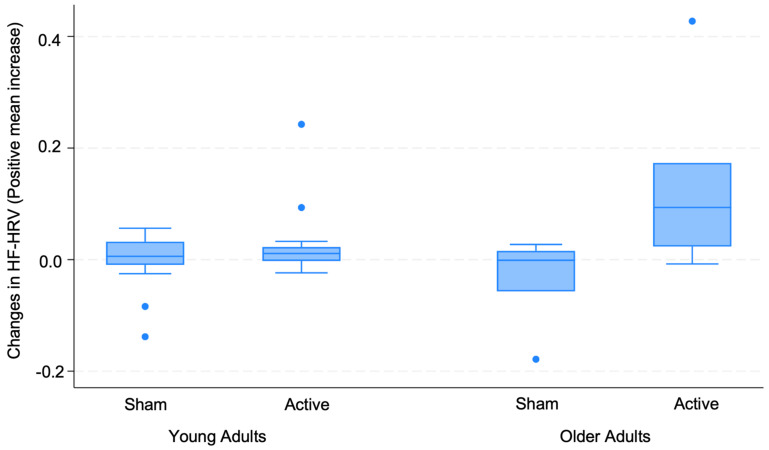
Descriptive comparison of high-frequency heart rate variability changes between young and older adults under sham and active taVNS conditions.

The variables mentioned in the outcome measure, such as age and sex, were tested as covariates in a linear regression model to control for their potential confounding effects on the relationship between taVNS treatment and HF_DIFF. Subsequently, a linear regression model was constructed to understand whether age or sex acted as moderators of the taVNS effect; thus, we included them as interaction terms. For context, among patients over 60 years old, males constituted 69.23% and females made up 30.77%. We found that the interaction term between age and treatment group was statistically significant (*p* = 0.02), indicating that age is a significant effect modifier. This suggests that age not only influenced the outcome but enhanced the efficacy of the active taVNS as compared to the sham, with the effect becoming more pronounced in older participants (Figure 4). On the other hand, the interaction term involving sex was not significant (*p* = 0.875), indicating that sex does not modify the taVNS effect on HF-delta (Table 2).

### 3.4. Correlation

When performing a Pearson correlation between the changes in the HRV variables, a strong positive correlation was observed between SDNN_diff_value and RMSSD_diff_value (r = 0.8409, *p* < 0.05), as well as a strong positive correlation observed between RMSSD_diff_value and PNN50_diff_value (r = 0.8231, *p* < 0.05). There is no other strong positive or negative relationship highlighted by this analysis.

### 3.5. Adverse Effects

Participants were asked about side effects at the end of the stimulation period. No serious or unforeseen adverse events were documented. Only one participant in the active group reported transient tingling behind their left ear at the onset of stimulation, dissipating within the subsequent five minutes. The session concluded as planned, and no notable group distinctions were observed (sham taVNS: 0%; active taVNS: 0.04%; Pearson’s chi-squared test: χ^2^(1) = 1.0233, *p* = 0.312).

### 3.6. Successful Blinding

We used a successful blinding questionnaire, where participants were queried about their assigned groups at the trial’s conclusion. Our findings revealed that 91% (40 individuals) provided incorrect responses regarding their group assignment, while only 9% (4 individuals) accurately identified their group. This clearly indicates the success of the blinding process.

## 4. Discussion

Our study showed that one single session of taVNS modulates HRV, with higher changes in HF after active stimulation, indicating a shift in cardiac autonomic function toward parasympathetic predominance in healthy subjects. There was also a significant interaction with age, which suggests that older subjects showed a greater modulatory effect of taVNS on this metric.

The general consensus is that when at rest, parasympathetic outflow is mostly reflected by HRV in the time domain, and HF-HRV is frequently used as a proxy for parasympathetic activity during spontaneous breathing [42]. Many organs and sensory areas in the outer ear and external auditory canal have been shown to interact, with the vagus nerve acting as a mediator. Examples of these interactions include the auriculo-cardiac reflex with cardiac inhibition, which can lead to syncope, and the ear-vomiting reflex [43]. Clancy et al. (2014) [23] found that stimulating the ear’s tragus with specific electrical pulses can shift the balance of the heart’s autonomic control toward the parasympathetic tonus. This effect was confirmed by a reduction in muscle nerve activity linked to stress, suggesting the potential of taVNS to influence heart rate and promote relaxation.

The VN is the longest of the cranial nerves, made up of 80% afferent fibers and 20% efferent fibers, and has an intrinsic role in regulating the homeostatic parameters of the cardiovascular and respiratory systems [44]. The auricular branch of the vagus nerve extends from the main bundle of the vagus nerve innervating the human ear, with most of its afferent projections to the brain located in the cymba conchae and tragus [45]. The current fMRI evidence of the vagus afference shows that when compared to earlobe (control) stimulation, cymba conchae stimulation significantly activated the central vagal projections, including the ipsilateral NTS, the bilateral trigeminal nuclei, the dorsal raphe, the locus coeruleus, the contralateral parabrachial area, the amygdala, and the nucleus accumbens, and from there to upstream cortical projections [18]. Based on that, stimulating the cymba conchae is able to activate brain areas which also control the vagal efferent. The insula, cingulate cortex, frontal and prefrontal cortices, hippocampus, thalamus, striatum, and amygdala showed the strongest correlations with HRV activity. Additionally, strong and mostly favorable relationships were found between HRV and brain area connectivity in the prefrontal cortex, cingulate cortex, and amygdala. Studies have shown intricate multi-level connections and support the idea that the brain and heart are connected by both structural and functional networks [46].

Regarding our stimulation site, a human auricle dissection study demonstrated that the cymba conchae and tragus are the regions with the most concentrated auricular branch of the vagus nerve projections [47]. Accordingly, we stimulated the conchae and used the HRV as one of the surrogates for measuring changes after the stimulation. We observed that the parameter that was more indicative of parasympathetic activity (HF-HRV) showed a significant increase in the active group. In our protocol, auricular vagus stimulation was performed bilaterally. Our recent meta-analysis showed that no cardiac problems were observed, and bilateral taVNS did not considerably raise the risk of unfavorable cardiovascular events [48], indicating that activation of the auricular vagus nerve stimulates more afferent fibers, which in turn produces an amplified effect on the central nervous system, rather than directly affecting the heart.

HRV is an accurate way to determine the health of the autonomic nervous system and offers vital information about its operation; low variation is present in sympathetic activation, such as stress conditions, with high variation in parasympathetic tonus, such as at rest or in relaxed conditions. Low HRV may indicate heart issues, whereas high HRV often means better health [49]. It is a useful tool for measuring the nervous system’s reaction to environmental, emotional, and cognitive stimuli. Research indicates that, in healthy subjects, a higher HRV indicates better adaptability and a more favorable state of recovery, whereas a lower HRV indicates stress and a less favorable state of recovery [49].

Research suggests that aging and older age are linked to reduced HRV. According to research, the 24 h average of 5 min segments, or rMSSD, decreases by about 3.6 milliseconds every ten years, reinforcing the increase in sympathetic tone with aging [50]. Furthermore, the Cardiovascular Health Study showed that the age range between 65 and 74 years was when the frequency-domain HRV values declined the most [51]. Our study showed that participants over 60 years old responded better to taVNS compared to younger participants, regardless of sex. This highlights that the higher sympathetic dominance that occurs with aging could be rebalanced using taVNS.

Increases in sympathetic neural activity and decreases in parasympathetic neural activity, as hallmarks of aging, are linked to modifications in autonomic nervous system function [52]. Aging also brings about important anatomical changes in the brain [53]. While more dorsal, lateral, and superior parts of the brain exhibit a higher loss in thickness with age, the more ventral regions, such as the ACC and the vmPFC, appear to remain generally maintained. Additionally, it has been proposed that in certain brain regions, there may be age-invariant correlations between HRV and cortical thickness [46]. According to the findings of a study by Yoo et al. [54], there may be age-constant correlations between cortical thickness in more ventral brain regions, like the lateral OFC (which is less associated with age-linked declines in cortical thickness) and HRV. Recently, studies have linked vagal tone and the anatomical–molecular pathways of cellular senescence and age-related disease. For instance, according to Ask and Sütterlin (2022) [55], prefrontally controlled vagal neuroimmunomodulation is linked to telomere length, hence validating the Neuro-Immuno-Senescence Integrative Model and introducing a potential area of study.

In summary, autonomic alterations can have a negative impact on gut, heart, and emotional health. They may also contribute to a number of age-related illnesses that are becoming more common, such as hypertension, heart failure, and depression. In order to improve autonomic function in healthy individuals, taVNS is a promising therapeutic. For example, research found that 15 min of taVNS elevated HRV and enhanced spontaneous heart baroreflex sensitivity in young, healthy participants, increasing parasympathetic activity [56]. Even with several studies in the area, there is still a lack of evidence regarding the acute effects of taVNS on HRV, and most of the findings are conflicting. For instance, Antonino et al. (2017) [26] and Clancy et al. (2014) [23] revealed a substantial decrease in the low-frequency/high-frequency ratio (LF/HF) in active taVNS compared to sham, indicating a rise in HRV. On the other hand, there were no differences in HF-HRV. taVNS also raised LF and LF/HF after extended stimulation (35 min), while short-term (10 min) stimulation had no effect on LF/HF [57]. This suggests that the duration of stimulation can have a possible adaptation effect depending on the metric of HRV. In the study of Geng et al. (2022) [22], a notable shift in parasympathetic activity was seen in the high-frequency domain for brief (5 min) taVNS stimulation, with an additional improvement in the measurements of RMSSD and SDRR reported in healthy young persons in a recent work [57]. Lately, a Bayesian random effects meta-analysis with 16 studies suggested that HRV is not a robust biomarker for acute taVNS [58]. Interestingly, the investigations included contrasted various designs, stimulation targets, and parameters, which may impact how the findings are interpreted.

Furthermore, the correlations observed in our study, using the differences in HRV values before and after taVNS, suggest that taVNS consistently influences HRV. This is particularly evident in the enhancement of parasympathetic modulation, as indicated by the correlation between the changes in RMSSD and PNN50, and implies a broader regulatory effect on autonomic balance, reflected in the positive correlation between the changes in RMSSD and SDNN.

Another notable insight from our results is that not only taVNS was able to modulate HRV, but older subjects presented greater changes in HF after taVNS compared to young subjects. Since baseline HRV declines with age [51], taVNS could be more beneficial for older recipients because it causes greater increases in HRV in those with lower baseline HRV. Based on that and the fact that taVNS has a safe profile, portability, and usability, the technique shows potential to preserve function, health, and well-being as well as to avoid or lessen the impact of age-related impairments.

While our study provides critical insights into HRV metrics following taVNS, it is important to acknowledge inherent limitations, notably the homogeneity of our sample, which can limit the generalizability of our findings. A second limitation is that in our study, we conducted only one session of intervention without a longer follow-up, which makes it more difficult to measure the effect of uncontrolled factors like different session times and times of HRV measurement, menstrual cycle stages in female participants, and exercise load for each participant, all of which could affect the results. Nonetheless, it is noteworthy that it was possible to observe and measure the changes in HRV metrics following the intervention.

## 5. Conclusions

Our research demonstrated that a single session of bilateral taVNS induces changes in HRV, particularly with increased HF components following active stimulation. This indicates a shift in cardiac autonomic function toward parasympathetic predominance in healthy individuals. Additionally, a noteworthy interaction with age was observed, indicating that older subjects exhibited a more pronounced modulatory effect of taVNS on this metric.

## Figures and Tables

**Table 1:** Demographic and baseline clinical characteristics (n = 44).

Variables	Sham Mean or % (SD)	Active Mean or % (SD)	Total Mean or % (SD)	*p*-Value
**Sample size**	22	22	44	
**Age (year)**	40.8 (17.8)	41.7 (22.8)	41.3 (20.2)	0.88
**Sex (female, %)**	54.5	68.2	61.3	0.53
**Ethnicity (%):**				0.22
**Non-Hispanic**	77.3	90.9	84.1	
**Hispanic**	22.7	9.1	15.9	
**Race (%):**				0.37
**American Indian or Alaska Native**	0.0	0.0	0.0	
**Asian**	31.8	13.6	22.7	
**Black or African American**	4.5	9.1	6.8	
**White**	50.0	63.6	56.8	
**Other**	9.1	13.6	11.4	
**Unknown or not reported**	4.5	0.0	2.3	
**Education level (%):**				0.55
**High school**	9.1	18.2	13.6	
**College**	40.9	45.5	43.2	
**Higher than college**	50	36.4	43.2	
**HR**	69.3 (9.5)	71.1 (9.2)	70.2 (9.3)	0.55
**LFs**	0.1 (0.1)	0.1 (0.0)	0.1 (0.0)	0.36
**HFs**	0.1 (0.1)	0.1 (0.1)	0.1 (0.1)	0.55
**LF/HF ratio**	0.9 (0.3)	1.0 (0.7)	0.9 (0.5)	0.40
**SDNN**	54.2 (27.3)	44.5 (24.6)	49.4 (26.1)	0.24
**RMSSD**	39.9 (27.2)	32.5 (27.0)	36.2 (27.0)	0.39
**pNN50**	18.1 (15.5)	15.6 (21.8)	16.9 (18.5)	0.70

HFs: high frequencies; LFs: low frequencies; SDNN: standard deviation of the N-N intervals; RMSSD: root mean square of the difference between successive R-R intervals; pNN50: percentage of R-R intervals that differed more than 50 ms.

**Table 2:** Adjusted treatment comparison.

-	Sham taVNS (*n* = 18) (Mean ± SD)	Active taVNS (*n* = 20) (Mean ± SD)	Unadjusted Effects Coef., 95% CI, *p*-Value	Adjusted Effects * Coef., 95% CI, *p*-Value
HF difference	0.1 ± 0.2	0.6 ± 0.3	1.95, (0.33–3.57), 0.02	2.01, (0.33–3.69), 0.02

* adjusted by age.

## Data Availability

Data generated or analyzed during this study are available from the corresponding author upon reasonable request.

## Supplementary Materials

The following supporting information can be downloaded at https://www.mdpi.com/article/10.3390/jcm13144267/s1: Table S1: Parameters and details based on the international consensus for reporting taVNS [59].
