# Supplementary material for: Age as an Effect Modifier of the Effects of Transcutaneous Auricular Vagus Nerve Stimulation (taVNS) on Heart Rate Variability in Healthy Subjects
Source: J Clin Med. 2024 Jul 22;13(14):4267. doi: 10.3390/jcm13144267 (PMC11278058; doi:10.3390/jcm13144267)
Supplement: Supplementary file 1 [file jcm-13-04267-s001.zip › jcm-3069462-supplementary.pdf]

Table S1. Parameters and details based on the International consensus for reporting taVNS [59]

**Manufacturer/name/version**	Neurive Co., Ltd / Healaon / 1.00.001
**Stimulation site**	Cymba Concha / Cavum Concha
**Electrode composition**	Polymer with carbon
**Current intensity (mA)**	0 ~ 2.5 (1: 0.5 mA; 2: 1mA; 3:1.5mA; 4:2.0 mA; 5:2.5 mA)
**Pulse width (μs)**	200
**Frequency (Hz)**	30
**Waveform descriptions:**	Square wave shape with anode (Cavum Concha) and cathode (Cymba Concha)
**Pulse shape and burst/non-burst stimulation**	bidirectional square wave, non-burst stimulation
**Voltage (mV)**	Voltage-controlled stimulation (1: 18.4 V; 2: 24 V; 3: 28.8 V; 4: 34.4 V; 5: 39.2 V)
